# Evaluating Humanisation in Intensive Care Nursing Practice: An Exploratory Study Using the HUMAS Scale

**DOI:** 10.1111/jep.70691

**Published:** 2026-10-22

**Authors:** Ángela Vázquez Villar, María José Ferreira Díaz

**Affiliations:** ^1^ School of Nursing University of Santiago de Compostela Lugo Spain

**Keywords:** critical care nursing, humanisation, intensive care units, nurse–patient relations, person‐centred care, relational care

## Abstract

**Background:**

Humanised care is central to nursing practice, yet sustaining relational and emotionally responsive care may be particularly challenging in intensive care environments characterised by technological complexity, clinical instability and repeated exposure to suffering. Humanisation should therefore be understood not as a single professional attribute, but as a multidimensional and context‐dependent aspect of nursing practice.

**Aim:**

To explore humanisation‐related dimensions among nurses working in an adult intensive care unit using the Healthcare Professional Humanisation Scale (HUMAS).

**Methods:**

An exploratory cross‐sectional study was conducted among 32 nurses working in an adult intensive care unit. Humanisation‐related dimensions were assessed using the Healthcare Professional Humanisation Scale (HUMAS). Descriptive analyses were used to examine scores across the dimensions evaluated by the instrument.

**Results:**

Sociability showed the highest mean score (4.4), followed by self‐efficacy (3.9), optimistic disposition (3.7), affection (3.6) and emotional understanding (2.9). These aggregated means describe a numerical pattern within this sample. In the absence of reported measures of variability or statistical comparisons, they cannot establish differences between dimensions or explain how nurses expressed these attributes in clinical practice.

**Conclusion:**

The findings provide an exploratory description of humanisation‐related dimensions among intensive care nurses and suggest that humanisation in this setting should be considered as a multidimensional construct. Further research using larger samples and more comprehensive statistical analyses is needed to examine these dimensions and their expression in intensive care practice.

## 1 Introduction

Humanised care is fundamental to nursing practice and involves recognising patients as persons whose physical, emotional, social and relational needs remain relevant alongside their clinical condition. It encompasses dignity, empathy, therapeutic communication, ethical sensitivity and attention to individual experiences, complementing scientific and technical competence [1, 2].

In intensive care, continuous monitoring and life‐sustaining interventions coexist with patients' vulnerability and dependence. Sedation, mechanical ventilation, delirium and impaired consciousness may limit communication, while families experience uncertainty and distress. Nurses combine physiological surveillance and complex interventions with the recognition of patients' needs, communication and family support. Humanising intensive care therefore involves integrating technically competent care with attention to patients' and families' experiences [3, 4].

The emotional and organisational context also warrants consideration. Empathy‐related guilt has been associated with burnout and compassion fatigue among nurses [5], while research on humanisation has considered communication, burnout and the conditions in which care is delivered [3, 6]. These perspectives provide background for studying humanisation‐related attributes, but do not establish how contextual factors influence scores in an individual sample.

The Healthcare Professional Humanisation Scale (HUMAS), developed and validated for nursing professionals, assesses five dimensions: optimistic disposition, sociability, emotional understanding, self‐efficacy and affection [7]. It offers a structured approach to describing professionals' self‐reported humanisation‐related attributes. Its scores should not be interpreted as direct measures of care quality, observed clinical behaviour or unit‐level performance.

Existing literature has addressed patient and family experiences, humanising practices and organisational approaches to intensive care [3, 4, 8]. Previous research using HUMAS has examined humanisation‐related attributes and their associations with communication and burnout in broader nursing samples [6]. The present study provides a setting‐specific descriptive account of the five HUMAS dimension means among nurses working in one adult ICU. Its contribution is limited to documenting these self‐reported attributes in a defined critical care setting and generating questions for subsequent research; it does not establish an ICU‐specific profile or differences from other nursing populations. Clinical demands and organisational conditions were not assessed, and their possible relevance is considered in the Discussion.

## 2 The Study

### 2.1 Aim

The aim of this exploratory study was to describe the aggregated mean scores of the five dimensions assessed by the Healthcare Professional Humanisation Scale (HUMAS) among nurses working in one adult intensive care unit: optimistic disposition, sociability, emotional understanding, self‐efficacy and affection. The study was not intended to evaluate individual professional performance or the overall quality of care provided by the participating intensive care unit.

## 3 Methods

### 3.1 Study Design

An exploratory descriptive cross‐sectional study was conducted using an anonymous self‐administered questionnaire. This design was selected to examine the profile of humanisation‐related attributes reported by intensive care nurses at a single point in time and to identify variation across the dimensions assessed by HUMAS. No intervention was implemented and no causal hypotheses were tested.

The study was reported in accordance with the Strengthening the Reporting of Observational Studies in Epidemiology (STROBE) guidelines [9].

### 3.2 Study Setting and Sampling

The study was conducted in an adult intensive care unit at a public university hospital in northwestern Spain. The unit provides specialised care for critically ill adults requiring continuous monitoring, advanced technological support and complex nursing interventions.

The nursing workforce comprised 68 professionals at the time of data collection. A non‐probability convenience sampling strategy was used, and all nurses who met the eligibility criteria were invited to participate. A total of 32 completed questionnaires were obtained, corresponding to a response rate of 47.1%. No formal sample‐size calculation was performed because the study sought to recruit eligible nurses from the participating intensive care unit. The final sample should therefore be regarded as exploratory rather than statistically representative of the wider intensive care nursing population.

### 3.3 Inclusion and Exclusion Criteria

Registered nurses were eligible to participate if they provided direct clinical care in the intensive care unit, had worked in the unit for at least 6 months and voluntarily agreed to participate. Nurses with fewer than 6 months of experience in the unit and those who did not complete the questionnaire in full were excluded.

The minimum period of intensive care experience was established to ensure that participants had sufficient exposure to the clinical, relational and organisational characteristics of the unit in which the study was conducted.

### 3.4 Instrument

Humanisation‐related attributes were assessed using the Healthcare Professional Humanisation Scale (HUMAS), a validated self‐report instrument developed for nursing professionals [7]. The scale comprises 19 items organised into five dimensions: optimistic disposition, sociability, emotional understanding, self‐efficacy and affection. The original Spanish‐language version of HUMAS was administered without modification to its items, response options or scoring procedure [7].

Items are rated using a 5‐point Likert response scale. Items assessing optimistic disposition, sociability, emotional understanding and self‐efficacy are scored from 1 (never) to 5 (always). The negatively worded items comprising the affection dimension are scored in the opposite direction, from 1 (always) to 5 (never), in accordance with the original HUMAS procedure. Higher scores therefore indicate a more favourable result across all five dimensions.

The original validation study supported the factorial validity of HUMAS and reported satisfactory internal consistency across its dimensions, with Cronbach's alpha coefficients ranging from 0.85 to 0.89 [7]. The instrument was selected because its multidimensional structure enables different personal, emotional and relational attributes relevant to humanised nursing care to be examined separately rather than reduced to a single global assessment. These coefficients refer to the original validation sample, not to the participants in the present study. Internal consistency was not estimated for our sample because individual item responses were not reanalysed for this manuscript.

A separate questionnaire collected sociodemographic and professional characteristics, including sex, age group and years of intensive care experience. To preserve anonymity, these data were collected independently and could not be linked to individual HUMAS responses.

### 3.5 Data Collection

Data were collected between April and early May 2025 using an anonymous online questionnaire administered through Google Forms. A link to the questionnaire, accompanied by information about the purpose of the study and conditions of participation, was distributed through an existing professional messaging channel used by nurses within the unit.

Participation was voluntary, and no financial or professional incentives were offered. Nurses could decide whether to access and complete the questionnaire without notifying the researchers or clinical managers. The questionnaire did not request names, identification numbers, email addresses or other directly identifying information.

HUMAS was administered first. Sociodemographic and professional characteristics were subsequently collected through a separate anonymous form. Because no identifier was used to connect the two forms, these characteristics were used only to describe the participating group and could not be associated with individual HUMAS scores.

### 3.6 Data Analysis

Data were analysed using Microsoft Excel. Descriptive statistics were used to summarise participant characteristics and the five HUMAS dimensions. Categorical variables were reported using frequencies and percentages, and the available dimension scores were summarised using arithmetic means.

No inferential statistical analyses were performed. Sociodemographic and professional information was collected separately from HUMAS responses and could not be linked at the individual level. Consequently, associations between participant characteristics and humanisation‐related dimensions could not be examined.

The individual HUMAS responses were retained by the student researcher as part of the original undergraduate project and analysed in Microsoft Excel for the dissertation. The present manuscript reports the five dimension means from that dissertation. We checked these values against the completed dissertation and its presentation, and they agreed. The individual responses were not reanalysed for the present manuscript; accordingly, measures of dispersion, confidence intervals, sample‐specific internal consistency and item‐level results are not reported. Access to individual responses was restricted to the undergraduate dissertation to protect participants' confidentiality and minimise potential identification risks.

The analysis was exploratory and descriptive. The five aggregated dimension means were reported descriptively. Individual‐level responses were not reanalysed for this manuscript; dispersion measures and statistical comparisons between dimensions are therefore not reported. No statistical or clinical significance was inferred from the numerical ordering of the means.

### 3.7 Ethical Considerations

The study was conducted in accordance with the ethical principles of the Declaration of Helsinki and applicable Spanish data‐protection legislation. The project was undertaken within an academic framework and involved an anonymous survey of nursing professionals, with no clinical intervention and no collection of patient data, clinical records, biological samples or directly identifiable personal information.

Authorisation to conduct the study and distribute the questionnaire was obtained from the nursing management of the participating hospital before data collection. All participants received information about the purpose of the study, the voluntary nature of participation, the anonymous handling of responses and the intended academic and scientific use of the aggregated findings. Informed consent was obtained from all participants before questionnaire completion.

### 3.8 Patient and Public Involvement

Patients and members of the public were not involved in the design, conduct, analysis or reporting of this study. Study participants were registered nurses providing clinical care in the intensive care unit.

## 4 Results

### 4.1 Characteristics of Participants

A total of 32 nurses participated in the study, corresponding to 47.1% of the 68 nurses employed in the intensive care unit at the time of data collection. Most participants were women (*n* = 26, 81.3%), while six were men (18.7%). Almost three‐quarters of participants were younger than 35 years (*n* = 23, 71.9%), and the same proportion had fewer than 10 years of intensive care experience (*n* = 23, 71.9%). Participant characteristics are presented in Table 1.

**Table 1:** Characteristics of participants (*n* = 32).

Characteristic	Category	*n*	%
Sex	Women	26	81.3
	Men	6	18.7
Age	< 35 years	23	71.9
	35–50 years	6	18.7
	> 50 years	3	9.4
Intensive care experience	< 10 years	23	71.9
	10–20 years	7	21.9
	> 20 years	2	6.2

*Note:* Sociodemographic and professional characteristics were collected independently from HUMAS responses and could not be linked to individual dimension scores. Percentages may not total 100 because of rounding.

### 4.2 Humanisation‐Related Dimension Profile

Mean scores were examined across the five dimensions assessed by HUMAS. Sociability showed the highest mean score (*M* = 4.4), followed by self‐efficacy (*M* = 3.9), optimistic disposition (*M* = 3.7) and affection (*M* = 3.6). Emotional understanding showed the lowest mean score (*M* = 2.9). The difference between the highest‐ and lowest‐scoring dimensions was 1.5 points.

The observed mean for sociability was numerically highest and that for emotional understanding was numerically lowest (Figure 1). This ordering describes the five aggregated values reported for this sample. Measures of dispersion and confidence intervals are not reported. Consequently, these means cannot show the distribution of individual scores or the precision of the estimates, and their numerical ordering does not establish statistically demonstrated differences between dimensions.

**Figure 1:**
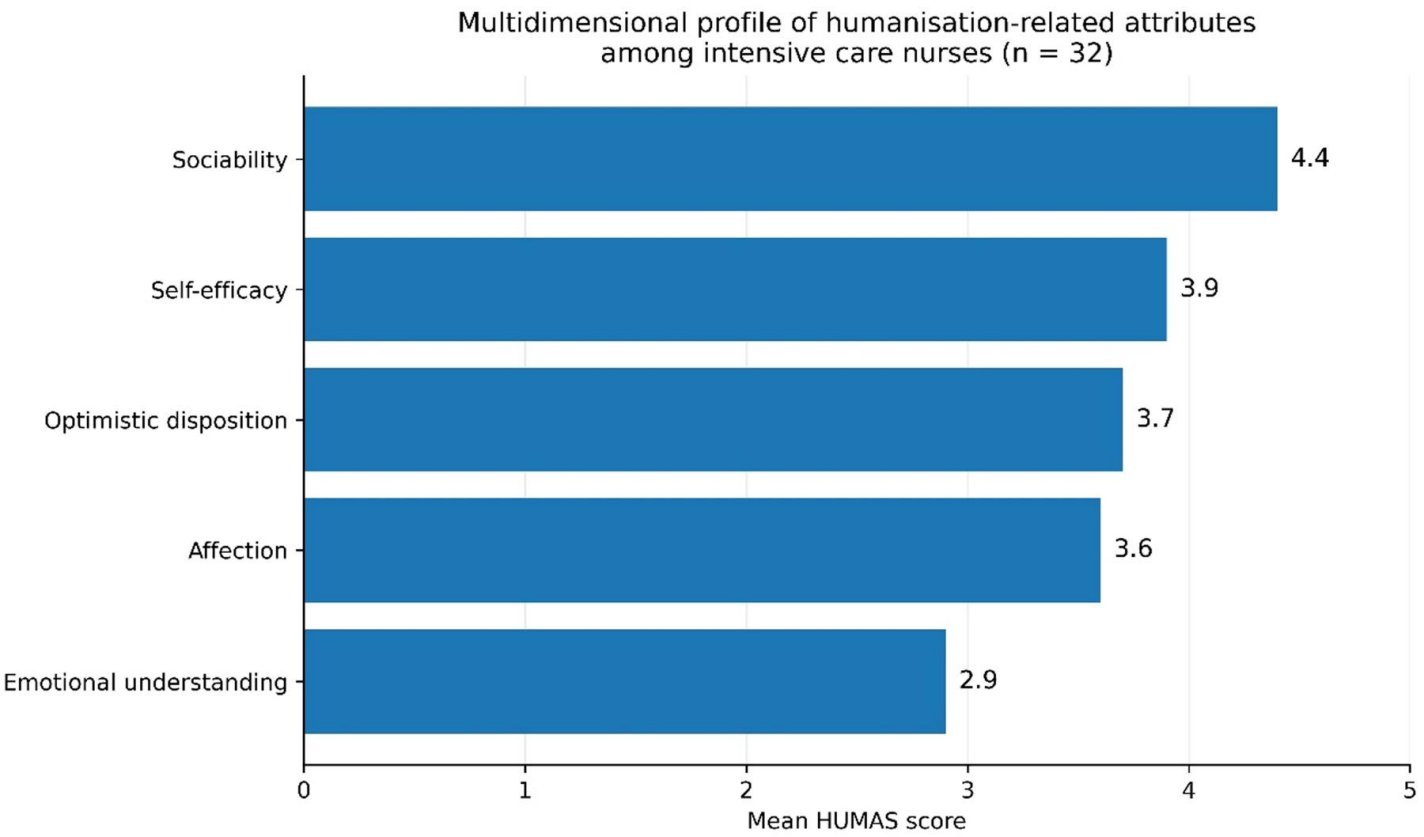
Mean scores of the five HUMAS dimensions among intensive care nurses (*n* = 32). Possible mean scores for each HUMAS dimension range from 1 to 5. Higher scores indicate higher levels of the corresponding HUMAS attribute. Items comprising the affection dimension are reverse‐scored in accordance with the original HUMAS scoring procedure. Values represent aggregated mean scores.

Because sociodemographic and professional characteristics were collected independently from HUMAS responses, dimension scores could not be examined according to sex, age or intensive care experience. No inferential comparisons between dimensions or participant subgroups were therefore performed. The results represent an exploratory descriptive profile of the participating nurses and should not be interpreted as an assessment of individual professional performance or the overall quality of humanised care within the unit.

## 5 Discussion

This exploratory study reports the aggregated mean scores of five HUMAS dimensions among nurses working in one intensive care unit. Sociability had the highest observed mean and emotional understanding the lowest, with the other three dimensions falling numerically between them. These values describe this sample; without reported measures of variability or statistical comparisons, they do not establish differences between dimensions or show how individual nurses practise humanised care. Nevertheless, examining the dimensions separately is consistent with conceptual accounts of humanising intensive care as a complex phenomenon involving professionals, patients, families and organisational conditions [3].

Staffing, workload, leadership, continuity of care and professional support were not assessed in this study. References to these factors in the following discussion therefore concern possible contextual influences identified in previous literature; they should not be interpreted as explanations for the observed HUMAS dimension means.

### 5.1 Relational Openness and Emotional Understanding: Different Dimensions of Humanised Care

Within the HUMAS framework, sociability reflects openness to interpersonal relationships and prosocial interaction, whereas emotional understanding concerns the recognition and interpretation of emotional states [7]. They are conceptually distinct dimensions of the instrument. In the present sample, their aggregated means were numerically different, but the available data cannot establish a reliable between‐dimension difference or its clinical meaning.

This distinction acquires particular significance in intensive care. Critically ill patients may experience impaired consciousness, mechanical ventilation, delirium, pain or difficulty communicating, circumstances in which recognising the person's experience beyond physiological parameters becomes particularly important [10]. Under these conditions, nurses must combine technically complex interventions with the interpretation of verbal and non‐verbal needs, communication and support for patients and relatives. Evidence from critical care research has identified communication, trust, information sharing and meaningful patient and family involvement as central to person‐centred care [4], while a scoping review of humanisation in intensive care described humanising care as encompassing holistic care, professional attitudes and an organisational ideal involving the wider healthcare system [3].

Relational availability should therefore not be assumed to imply equivalent capacity across all emotional processes. Establishing a close and supportive relationship may differ from recognising and interpreting complex emotional states in patients who cannot communicate conventionally or in relatives experiencing acute distress. The comparatively lower emotional understanding score in the present study should consequently not be interpreted as evidence of deficient empathy or less humanised clinical behaviour. HUMAS assesses self‐reported attributes, and the available aggregated data cannot establish how these attributes were enacted in individual encounters or experienced by patients and families.

This interpretation also challenges an overly simplified association between humanisation and visible interpersonal warmth. Recent empirical evidence examining dehumanisation among nurses has similarly highlighted the relevance of emotional responses, professional self‐perception and the clinical environment, suggesting that humanised practice cannot be inferred from interpersonal behaviour alone [11]. Rather, humanisation involves several related but distinct processes—including relational availability, emotional recognition, professional confidence and regulation of emotional involvement—which may coexist differently within the same professional and clinical context. Research using HUMAS in a larger sample of Spanish nurses has similarly demonstrated that humanisation dimensions form discernible profiles and are related to communication and occupational outcomes, supporting the value of considering these attributes multidimensionally rather than as an undifferentiated construct [6].

### 5.2 The Emotional Work of Humanised Intensive Care

Humanised intensive care requires emotional presence, but emotional presence should not be equated with unrestricted emotional involvement. Intensive care nurses repeatedly encounter suffering, deterioration, uncertainty, death and ethically complex situations while remaining responsible for technically demanding and time‐sensitive care. Humanisation therefore involves not only responsiveness to the experiences of patients and families, but also regulation of professionals' own emotional responses.

The affection dimension is particularly relevant to this distinction. HUMAS assesses affection through negatively worded emotional responses that are reverse‐scored, making interpretation more complex than simply assuming that greater emotional involvement represents greater humanisation [7]. Being emotionally affected by another person's suffering is not inherently incompatible with professional functioning, just as emotional distance cannot automatically be interpreted as successful emotional regulation.

Evidence concerning empathy‐related emotional burden reinforces this complexity. Duarte and Pinto‐Gouveia [5], in a study of 298 nurses, found that empathy‐based guilt was associated with burnout and compassion fatigue and that forms of guilt characterised by excessive responsibility for others' well‐being could compromise professional quality of life. Similarly, Molero Jurado et al. [6] identified relationships between humanisation profiles, communication and components of burnout among Spanish nurses. These findings do not imply that empathy or emotional involvement is harmful; rather, they indicate that the relationship between relational responsiveness and professional well‐being depends partly on how emotional experiences are processed and supported.

The present study did not measure burnout, compassion fatigue or emotional distress, and no such outcomes can be inferred from the HUMAS profile. Nevertheless, considering affection alongside emotional understanding, sociability and self‐efficacy highlights why humanisation cannot reasonably be reduced to a demand for professionals to become progressively more emotionally available. Sustaining compassionate relationships may require an appropriate balance between openness to another person's experience, emotional regulation and professional boundaries.

Professional well‐being is therefore relevant to humanised care rather than external to it. Supporting nurses in processing emotionally demanding encounters may contribute to sustaining relational engagement, particularly when such support is embedded within broader organisational conditions rather than framed solely as an individual responsibility [3, 6].

### 5.3 Beyond the Individual Nurse: Humanisation As Organisational Practice

The multidimensional interpretation of humanisation also requires moving beyond the individual nurse. Humanising intensive care has been conceptualised not only in terms of professional attitudes or interpersonal behaviours, but as an organisational ideal involving patients, relatives, professionals and healthcare systems [3]. From this perspective, clinical environments may facilitate or constrain the expression of individual relational capacities. This represents a contextual hypothesis informed by previous literature, rather than an explanation established by the present study.

Relational care requires time, continuity and attentional capacity. The capacity to sustain relational care may also be influenced by nursing care complexity, as care demands can vary substantially even among medically similar patients [12, 13]. Such variability could affect the time and attention available for relational care, although this potential relationship was not assessed in the present study. Standardized nursing data offer a structured approach to characterising these care demands in future research [14]. In intensive care, these resources coexist with continuous surveillance, complex technologies, rapidly changing physiological conditions and competing clinical priorities. Organisational factors, including staffing, workload, continuity of care, physical environments, visiting practices, leadership and professional support, may influence opportunities for communication, patient‐centred care and sustained relational presence [3, 15]. Humanisation initiatives have consequently extended beyond individual professional training to encompass communication practices, family participation, environmental changes and organisational models of care [3, 16]. Recent empirical evidence reinforces the importance of this organisational context. Among intensive care nurses, Seo et al. [17] found that the nursing work environment moderated the relationship between clinical judgement ability and person‐centred care, with more favourable work environments strengthening this relationship. This finding supports the view that professional capacities do not operate independently of the conditions in which nurses practise.

The present findings cannot establish whether any of these factors contributed to the observed HUMAS profile. In particular, the lower emotional understanding score cannot be attributed to workload, staffing, professional experience or organisational conditions because these relationships were not examined. Equally, the comparatively high sociability score cannot demonstrate that relational openness was consistently translated into therapeutic encounters. Maintaining this distinction between empirical findings and contextual interpretation is important in avoiding the individualisation of a phenomenon that existing literature describes as multidimensional and organisationally situated.

Communication training, emotional‐awareness interventions and reflective practice may strengthen individual capacities, but interventions directed exclusively towards professionals risk being insufficient if clinical conditions restrict their application. Humanisation strategies should therefore consider professional development alongside organisational conditions that support communication, continuity, emotional processing and patient and family involvement.

Humanised intensive care thus depends on nurses but cannot reasonably be made the responsibility of nurses alone. Relational and emotional capacities are exercised within systems that may enable, constrain or sustain them, making humanisation a shared professional and organisational responsibility.

### 5.4 Clinical Interpretation of Humanisation Profiles: What Multidimensional Measurement Can and Cannot Show

The findings also raise an important question concerning the clinical interpretation of multidimensional humanisation profiles. HUMAS was developed as a multidimensional measure encompassing optimistic disposition, sociability, emotional understanding, self‐efficacy and affection, with evidence supporting its factorial structure and reliability in nursing professionals [7]. Its multidimensional structure is particularly valuable because it allows potentially divergent components of humanisation to remain visible.

The reported dimension means share a numerical range of 1–5, with affection scored in the opposite direction in accordance with the HUMAS procedure. However, a common numerical range does not establish psychometric equivalence across subscales, which assess distinct attributes and differ in item content and wording [7]. Their numerical ordering is therefore presented descriptively and should not be interpreted as demonstrating that nurses possess more of one capacity than another.

Reporting the five dimension means separately preserves descriptive information that would be obscured by a single global score. In this sample, the observed mean for sociability was numerically higher than that for emotional understanding. However, without measures of variability or statistical comparisons, this numerical ordering cannot establish differences between dimensions or demonstrate a non‐uniform humanisation profile. Previous research using HUMAS has examined its constituent dimensions and their relationships with communication and burnout [6].

Measurement nevertheless has clear boundaries. Self‐reported HUMAS scores cannot establish how nurses behave in particular clinical encounters, how patients and families experience those encounters or which organisational circumstances facilitate or constrain relational practice. In the present study, the absence of an individual‐level reanalysis and the inability to link HUMAS responses with participant characteristics further restrict the interpretations that can be made. Scores should therefore not be used in isolation to label individual professionals or to classify the overall quality of humanisation within a clinical unit.

The value of a multidimensional profile may instead lie in the questions it generates. The observed numerical ordering of dimension means can generate questions for further enquiry, but cannot establish differences in nurses' underlying capacities or explain how emotional, relational and organisational aspects of nursing interact. Future studies combining HUMAS with qualitative accounts, patient and family perspectives, direct observation and measures of the clinical work environment could help determine whether apparently divergent profiles reflect individual characteristics, contextual pressures or interactions between them.

Future studies should adopt multicentre designs with larger samples and confidential participant codes linking individual HUMAS responses to demographic and professional characteristics. Such designs would permit subgroup comparisons and multivariable analyses, provided that sample size and data quality are adequate.

Future research should also examine whether variation in HUMAS dimensions, particularly emotional understanding, is associated with the complexity of nursing care encountered in clinical practice. Standardized nursing data, including nursing diagnoses and documented interventions, could help characterise patients' care needs and support analyses linking these needs with nurses' HUMAS scores and relevant clinical trajectories [14, 18]. Such studies would require linked nurse–patient or nurse–shift data to establish the care demands encountered by each participating nurse. Nursing care complexity was not measured in the present study and therefore cannot explain the observed dimension means.

Measurement, from this perspective, becomes a starting point for enquiry rather than an endpoint of evaluation. Used critically, HUMAS can make relational and emotional dimensions of nursing visible without reducing humanised care to a score or separating professional attributes from the clinical contexts in which they acquire meaning.

### 5.5 Strengths and Limitations

This study has several strengths. It examines humanisation within a real‐world intensive care environment in which technological complexity, emotional demands and relational care coexist continuously. The use of HUMAS, an instrument specifically developed and validated for nursing professionals, provided a theoretically grounded multidimensional framework encompassing optimistic disposition, sociability, emotional understanding, self‐efficacy and affection [7]. Rather than reducing these dimensions to a global judgement of humanisation, the present analysis reported the five aggregated means separately and interpreted their numerical ordering cautiously. This approach is consistent with broader understandings of humanised intensive care as a multidimensional phenomenon involving professionals, patients, families and organisational conditions [3].

Several limitations nevertheless constrain the interpretation of the findings. First, the study was conducted in a single adult intensive care unit and included a convenience sample of 32 nurses, representing 47.1% of the eligible nursing workforce. The small sample, single‐centre setting and potential non‐response bias substantially limit the generalisability of the findings. Nurses who participated may have differed from non‐participants in their interest in humanisation or in their self‐reported attributes; these differences could not be assessed. The reported means therefore provide a descriptive account of the participating sample and should not be considered representative of all nurses in the unit or of intensive care nurses more broadly.

Second, HUMAS is a self‐report measure. Responses may therefore have been influenced by social desirability and represent professionals' perceptions of their own relational and emotional attributes rather than directly observed clinical behaviour. The study did not include patient or family perspectives, direct observation or qualitative accounts that could triangulate these perceptions. Consequently, the findings cannot be interpreted as a direct assessment of the humanisation or quality of care delivered within the unit.

Third, sociodemographic and professional characteristics were collected separately from HUMAS responses to preserve anonymity, without a code allowing individual‐level linkage. This decision introduced a limitation at the study‐design stage: associations between age, sex or intensive care experience and HUMAS dimensions could not be examined, irrespective of the availability of the original responses. This is particularly relevant because 71.9% of participants were younger than 35 years and 71.9% had fewer than 10 years of intensive care experience. These characteristics describe the sample but cannot explain the observed emotional understanding or self‐efficacy scores. Future studies should use confidential participant codes to permit linkage while protecting participants' identities.

Finally, although the individual responses were retained as part of the original undergraduate project, they were not reanalysed for this manuscript. The five reported means agree with the completed dissertation and its presentation, but this comparison does not independently verify the original item scoring or calculations. Measures of dispersion, confidence intervals and sample‐specific internal consistency are not reported. Without measures of variability, the means cannot show whether participants' responses were closely clustered or widely dispersed, and different distributions of individual scores could produce the same mean. The precision of these estimates and the consistency of responses within each dimension therefore cannot be assessed from the reported summaries. The findings should therefore be interpreted descriptively.

These limitations reinforce the exploratory purpose of the study. The findings are not intended to establish causal explanations or to classify individual nurses or the participating unit. Their contribution lies in reporting descriptive scores that may inform future research into relational and emotional aspects of intensive care.

## 6 Conclusion

This exploratory study reports five aggregated HUMAS dimension means among nurses working in one intensive care unit. Sociability had the highest observed mean and emotional understanding the lowest. Without measures of variability or statistical comparisons, this numerical ordering cannot establish differences between dimensions or characterise individual nurses. The findings should be interpreted as descriptive and may inform questions for future research on the multidimensional nature of humanised care.

Future research should combine individual‐level HUMAS data with patient and family perspectives and measures of nursing care complexity and organisational conditions. Such designs could examine whether self‐reported attributes are associated with observed relational care and the circumstances in which it is delivered.

## Author Contributions

Ángela Vázquez Villar contributed to the conception of the original undergraduate research project, literature review, data collection and initial interpretation of the findings as part of her Final‐Year Undergraduate Nursing Project (Trabajo Fin de Grado, TFG). María José Ferreira Díaz supervised the original academic project and contributed to its conception, methodological oversight and interpretation. Both authors contributed to the development of the manuscript for scientific publication, critically reviewed its intellectual content and approved the final version. Both authors meet the International Committee of Medical Journal Editors (ICMJE) criteria for authorship and agree to be accountable for all aspects of the work.

## Funding

The authors have nothing to report.

## Ethics Statement

This study originated from a Final‐Year Undergraduate Nursing Project (Trabajo Fin de Grado, TFG) conducted during the 2024–2025 academic year. It involved an anonymous, voluntary survey of registered nurses working in an adult intensive care unit and did not involve patients, clinical interventions, access to medical records, biological samples or the collection of directly identifiable personal information.

Authorisation to conduct the study and distribute the questionnaire was obtained from the nursing management of the participating hospital before data collection. Participants received information about the purpose of the study, the voluntary nature of participation, the anonymous handling of responses and the intended academic and scientific use of the aggregated findings. Informed consent was obtained from all participants before questionnaire completion. The study was conducted in accordance with the principles of the Declaration of Helsinki and applicable Spanish data‐protection legislation.

## Conflicts of Interest

The authors declare no conflicts of interest.

## Data Availability

The individual‐level HUMAS data are not publicly available due to participant confidentiality and the potential risk of identification associated with the small sample from a single intensive care unit. The present manuscript reports aggregated dimension means derived from the completed undergraduate dissertation. Individual‐level data were not available for reanalysis for the present manuscript.
